# Formulation and evaluation of bilayer tablet for bimodal release of venlafaxine hydrochloride

**DOI:** 10.3389/fphar.2015.00144

**Published:** 2015-07-09

**Authors:** Munira M. Momin, Snehal Kane, Pooja Abhang

**Affiliations:** ^1^Department of Pharmaceutics, SVKM’s Dr. Bhanuben Nanavati College of PharmacyMumbai, India; ^2^Department of Pharmaceutics, Oriental College of PharmacyNavi Mumbai, India; ^3^RK UniversityRajkot, India

**Keywords:** natural polysaccharide, bilayer tablet, immediate release layer, bioadhesive layer, bimodal drug release, factorial design

## Abstract

The aim of the present research was to develop a bilayer tablet of venlafaxine hydrochloride for bimodal drug release. In the present investigation authors have tried to explore fenugreek mucilage (FNM) for bioadhesive sustained release layer. The attempt has been made to combine FNM with well studied bioadhesive polymers like hydroxy propyl methyl cellulose (HPMC), Carbopol, and Xanthan Gum. The formulations were evaluated for swelling Index, *ex vivo* bioadhesion, water uptake studies, *in vitro* drug release and dissolution kinetics was studied. Substantial bioadhesion force (2.4 ± 0.023 g) and tablet adhesion retention time (24 ± 2 h) was observed with FNM and HPMC combination at 80:20 ratio. The dissolution kinetics followed the Higuchi model (*R*^2^ = 0.9913) via a non-Fickian diffusion controlled release mechanism after the initial burst. The 3^2^ full factorial design was employed in the present study. The type of polymers used in combination with FNM (X1) and percent polymer replaced with FNM (X2) were taken as independent formulations variables. The selected responses, bioadhesion force (0.11–0.25 ± 0.023 g), amount of drug released in 10 h, Y_10_ (78.20–95.78 ± 1.24%) and bioadhesive strength, (19–24 ± 2 h) presented good correlation with the selected independent variables. Statistical analysis (ANOVA) of the optimized bilayer formulations showed no significant difference in the cumulative amount of drug release after 15 min, but significant difference (*p* < 0.05) in the amount of drug released after 1 hr till 12 h from optimized formulations was observed. The natural mucilage like FNM could be successfully incorporated into tablet with only 20% replacement with HPMC and it showed good bioadhesiveness and sustained drug release.

## Introduction

The new drug delivery system with better efficacy and safety with reduced dosing frequency and improved patient compliance is the current area of research by formulation development scientists. Tablet being most preferred dosage form for its ease of manufacturing and patient convenience is always a first choice of dosage form. The single layer tablets leads to frequent dosing and unpredicted drug plasma level for drugs with shorter half lives (Banker and Anderson, 1986; Ansel et al., 2011). Number of diseases like schizophrenia require immediate release of drug for instant effect to manage the panic attack at its presentation and then drug concentration has to be maintained for prolong effect of the drug. On the basis of requirement for such a disease conditions, the multilayered tablet concept has been utilized (Preeti, 2011). Such a tablet has a fast releasing layer and may contain bi- or triple layers to sustain the drug release. Bilayer tablets present a better choice where one layer provide immediate dose which is then maintains the plasma drug level by its controlled release layer of the tablet. Venlafaxine hydrochloride multilayered tablet has been developed by several scientists and reported earlier which is frequently prescribed for management of panic depression attacks. (Gohel and Bariya, 2009; Sharif et al., 2011; Niwa et al., 2013) Venlafaxine Hcl (VFX) is a new generation anti-depressant serotonin/noradrenalin reuptake inhibitor drug showing effective anti-depressant properties. It has a short biological half-life of 5 h. So, frequent administration is necessary to maintain its therapeutic concentration. This necessitates multiple daily dosing of VFX for maintenance of its plasma concentration within the therapeutic index. Venlafaxine Hcl (VFX) has site specific absorption for upper GI tract. Hence, all these properties make VFX an ideal candidate for sustained release and bioadhesive drug delivery system (Muth et al., 1991; Holiday and Benfield, 1995; Budavari, 1996; Parfitt, 1999; Makhijal and Vavia, 2002; Prasad et al., 2010).

Reports have been made for use of sodium starch glycolate (SSG), Cross carmellose, cross povidone, and Hydroxy propyl methyl cellulose (HPMC), chitosan, Xanthan Gum in the formulation of bilayer tablets as superdisintegrants and sustained release polymer, respectively, in the formulation of bilayer tablets (Kawtikwar et al., 2009; Reddy et al., 2010; Logidhasan et al., 2013)

In recent years natural polymers have been widely used because of their effectiveness and availability over synthetic polymers. Natural polysaccharides and dried mucilage have been reported as an emulsifying, suspending agent, binding agent, disintegrating agent, and as a sustained-release matrix. They have been utilized in variety of formulations like mucoadhesive, gastro retentive, colon specific drug delivery system etc., (Baveja et al., 1988; Ahuja et al., 1997; Rishabha et al., 2010; Pooja Abhang et al., 2012; Divani et al., 2013). Flax is an annual plant of the linaceae family is an annual herb of leguminosae family. This plant contains natural oligosaccharides (Fedeniuk and Biliaderis, 1994). Flax seeds produce high viscosity mucilage at low concentration levels. it has good water-holding capacities, owing to its marked swelling capacity and high viscosity in aqueous solution. The same reports have presented results for its application as a binder as well as bioadhesive matrix forming agent (Nerkar and Gattani, 2011, 2013; Pooja Abhang et al., 2012).

In the present study, a bilayer tablet for bimodal drug release in which one layer of immediate release and second layer of sustained release of VFX was designed. The sustained release layer was formulated using FNM in combination with HPMC K 100 M, Carbopol 934P, and Xanthan Gum. The immediate layer was formulated using superdisintegrants such as SSG, Cross-Carmellose sodium, and Cross-Povidone. In the present study a systematic approach has been adopted to study the effect of FNM in combination with a lowest possible level of well known bioadhesive hydrophilic polymers like, HPMC, Xanthan Gum, and Carbopol. The present formulation will meet the need of an antidepressant with shorter half life to immediately release the drug to control the panic attack and then maintain the plasma concentration for managing the further symptoms.

Statistical optimization techniques are frequently employed for the development of pharmaceutical formulations (Vinayagamkannan et al., 2003). A 3^2^ full factorial design is the simple experimental design with two variables studied at three levels. In the present study, the type of polymers used in combination with FNM (X1) and percent polymer replaced with FNM (X2) to achieve required bioadhesion and sustained release of VFX were taken as independent formulations variables. The amount of drug released in 10 h (Y_10_), bioadhesive strength and bioadhesion time were taken as the dependent response variables.

## Materials

Venlafaxine HCl VFX was received as a gift sample by Shree Pharmaceutical Ltd., Mehasana, Gujarat, India. Fenugreek seed was procured from local market of chembur, Mumbai, India. Hydroxypropylmethyl cellulose K 100 (HPMC) were obtained as a gift sample from Colorcon Asia Pvt. Ltd., Goa. Carbopol 934P, Xanthan Gum, SSG, cross carmellose sodium, cross povidone, Microcrystalline cellulose (MCC), magnesium stearate, and Talc were procured from S. D Fine Chemicals, Mumbai, India. Ethanol was obtained from Goggia and company, Mumbai, India. Goat stomach mucosa for determining bioadhesive strength was obtained from a local slaughter house of Mumbai, India. All other chemicals and reagents used were of analytical grade, and were used as received.

## Methods

### Isolation of Fenugreek Mucilage

The fenugreek seeds (*Trigonella foenum graceum L*.) used in study was procured from a local market in Mumbai, India. The seeds were authenticated by Professor Harshad Pandit, Khalsa College, Mumbai, India.

The isolation of mucilage of fenugreek seeds (FNM) was done by method described by Pooja Abhang et al. (2012). Briefly, 100 g of crushed fenugreek seeds was soaked in 500 ml of double distilled water for overnight and boiled at 80°C using water bath for 4 h with occasional stirring or till thick mass was obtained. It was kept aside at room temperature for 4 h with intermittent stirring and then kept aside for overnight below 20°C. The hydrated mucilage was separated by using muslin cloth. The mucilage was then precipitated with 300 ml of absolute alcohol. The precipitated mucilage was filtered using vacuum filtration. The filtered mucilage was then dehydrated with 200 ml of acetone. This treatment also removes any oil if present in hydrated mucilage. After filtration precipitated mass was dried in hot air oven at 50°C for 12 h. The dried mucilage was then powdered using mortar and pestle and passed through sieve 60.

### Dose Calculation According to the Half Life

The total dose of venlafaxine for the loading dose in immediate layer and maintenance dose in sustained release layer of bilayer tablet was calculated by following equation using available pharmacokinetic data (Karna, 2012).

$$\mathrm{Dt}=\mathrm{Dose}\;(1+0.\mathrm{693}\times t/t_{1/2})$$

Where, Dt = Total dose of drug

Dose = dose of immediate release part (37.5 mg)

*t* = Time (hours) during which the sustained release is desired (12 h)

*t*_1/2_ = Half-life of drug (5)

Dt=37.5 (1+0.693×12/5)=99.87mg

In the preparation of bilayer tablet, fraction of the drug in both the phase was adjusted as per USP requirement for initial drug release of 50–60%. Hence the present formulations contain total dose of 87.5 mg which includes 37.5 mg as a loading dose in immediate release layer and 50 mg is a maintenance dose in sustained release layer.

### Preparation and Characterization of Bilayer Tablets

To formulate bilayer tablets, fast-release, and sustained-release layers were initially prepared separately to study the dissolution profile of each layer with an objective of selecting the optimized combination of excepients for the formulations. The optimized formulation of each layer was then compressed to bilayer tablet and further *in vitro* drug dissolution data were compared with the marketed product.

### Formulation of the Immediate Release Layer

The dose in the formulation for immediate release layer was 37.5 mg. The immediate release tablet was prepared by blending venlafaxine hydrochloride uniformly with different type of super disintegrants (SSG, cross povidone and cross carmellose sodium) as per the formulae given in **Table 1**. The drug-superdisintegrant blend was then mixed with MCC using twin blender for 10 min. The final mass was lubricated with 0.5%w/w magnesium stearate and 0.5%w/w talc and compressed using Rotary Mini tablet press (Karnavati Pvt. Ltd., India) using 11 mm flat beveled punches. The tablets were evaluated for weight variation, thickness, friability, hardness, and disintegration time.

**Table 1:** Formulation design for immediate release tablets.

Ingredient	*B*_1_	*B*_2_	*B*_3_	*B*_4_	*B*_5_	*B*_6_
Venlafaxine(mg)	37.5	37.5	37.5	37.5	37.5	37.5
sodium starch glycolate (SSG) (%)	5	10	–	–	–	–
Crosspovidone (%)	–	–	5	10	–	–
Crosscarmellose (%)	–	–	–	–	5	10
Microcrystalline cellulose (MCC) (mg)	142.5	142.5	142.5	142.5	142.5	142.5
Total (mg)	200	200	200	200	200	200

### Formulation of the Sustained Release Layer

The sustained release layer of the tablet was prepared by wet granulation technique by mixing VFX uniformly with dried fenugreek mucilage (FNM) powder along with different proportion of HPMC K100 M, carbopol 934P, and Xanthan Gum as given in **Table 2**. Polyvinyl pyrolidone K 30 (3% w/v in IPA) was used as a binder. The wet mass was passed through 30# to obtain granules. The granules were dried at 60° in a tray drier. The granules of 30/60# size were lubricated with 1% w/w magnesium stearate. The sustained release granules were compressed using Rotary Mini tablet press (Karnavati Pvt. Ltd., India) equipped with 11 mm flat beveled punches. A constant tablet weight of 400 mg was maintained.

**Table 2:** Factorial design coded value layout of Venlafaxine HCl (VFX) sustained release layer.

Translation of coded levels in actual units			
Coded level	-1	0	+1
X1: Type of polymer	XNG	CBP	Hydroxy propyl methyl cellulose (HPMC)
X2 : % replacement with Fenugreek Mucilage (FNM)	20	40	60

### Optimization of the Sustained Release Layer Using 3^2^ Factorial Design

A 3^2^ full factorial design was applied was employed to systematically design and develop sustained release bioadhesive layer for bilayer VFX tablet. The type of polymers used in combination with FNM (X1) and percent polymer replaced with FNM (X2) to achieve required bioadhesion and sustained release of VFX were taken as independent formulations variables. The amount of drug released in 10 h (Y_10_), bioadhesive strength and bioadhesion time were taken as the dependent response variables. A statistical model incorporating interactive and polynomial terms was utilized to evaluate the resultant responses. The polynomial equation generated by this experimental design using Design Expert 7.1.6 software, State Ease Inc. is as follows,

(1) $$Y=b0+b1X1+b2X2+b12X1X2+{b11X1}^{2}+{b22X2}^{2}$$

where, Y is a response (dependent variable), b0 is an intercept, b1 to b33 are regression coefficients and X1 and X2 are independent formulation variables.

A total of nine batches were prepared as per the **Table 2**. Also, efforts were made to prepare and compare FNM-polymer blend sustained release tablet with tablets containing no FNM but only polymers (Carbopol, Xanthan Gum, HPMC). The purpose was to compare factorial batches for their drug release, bioadhesion, and retention time with that of formulations containing tablet without FNM (Batch F10–F12) as per **Table 3**.

**Table 3:** Factorial batches composition with dependent and independent variable values.

Batch	Independent formulations variables				Dependent variables		
	X1: Type of Polymer		X2: % FNM replaced		Y_10_	Bioadhesion (g)	Retention time (h)
	Coded value	Actual value	Coded value	Actual value			
**Factorial batches**			
F1	-1	XNG	-1	20	78.901	0.20	23
F2	-1	XNG	0	40	84.34	0.17	20
F3	-1	XNG	+1	60	87.61	0.20	20
F4	0	CBP	-1	20	87.239	0.24	24
F5	0	CBP	0	40	82.62	0.11	19
F6	0	CBP	+1	60	81.922	0.19	21
F7	+1	HPMC	-1	20	84.139	0.18	21
F8	+1	HPMC	0	40	89.395	0.21	21
F9	+1	HPMC	+1	60	95.32	0.23	22
**Non-factorial batches for comparison purpose**			
F10		XNG		100	71.2	0.25	26
F11		CBP		100	97.36	0.24	16
F12		HPMC		100	93.2	0.26	17

### Characterization of Granules

Prior to compression, granules were subjected to pharmacotechnical characterization. They were evaluated for tapped density, Carr’s index and angle of repose. Carr’s compressibility index was calculated from the bulk and tapped densities (Ansel et al., 2011) using a digital tap density apparatus (Electrolab Ltd, India).

### Compression of Bilayer Tablet

The bilayer tablet of venlafaxine was prepared using a Rotary Mini tablet press (Karnavati Pvt. Ltd., India) equipped with 11 mm beveled, flat punches. The die was initially filled with the weighed amount sustained release portion and were lightly compressed. Over this compressed layer, the required quantity of the fast release layer powder mixture was placed and compressed to obtain hardness of the tablet 6–7 kg/cm^2^. It was observed that table compressed at this force did not show any layer separation. The total weight of the tablet was kept constant, i.e., 400 mg for all formulation.

### Pharmacotechnical Evaluation of Venlafaxine Bilayer Tablets

The bilayer tablet of venlafaxine were evaluated for pharmacotechnical parameters like, hardness, friability, weight variation, thickness uniformity, and content uniformity as per the method described in official pharmacopeia (United States of Pharmacopoeia Convention [USP], 1999).

### *In Vitro* Dissolution Studies

Release of VFX was determined using a USP 24 (1999) six stage dissolution rate test apparatus 1 (Labindia Instruments Pvt. Ltd., India) at 50 rpm. The dissolution was studied using 900 mL of simulated gastric fluid (without enzyme, pH 1.2) for 10 h. The temperature was maintained at 37 ± 0.2°C. The sample (5 mL) was withdrawn at every 1 h time intervals and filtered through Whatman filter paper (Auroco Pvt. Ltd., Thailand) and replaced by an equal volume of dissolution medium. Samples were suitably diluted and analyzed for venlafaxine hydrochloride content at 224 nm (United States of Pharmacopoeia Convention [USP], 1999).

### Drug-Release Kinetics

In order to investigate the kinetics of drug release from the sustained release FNM layer of the bilayer tablets, the data of *in vitro* drug release were fitted to different models. The program was developed using PCP Disso software developed by Poona College of Pharmacy, India for zero order, first order, Higuchi, Hixson–Crowell, Korsmeyer–Peppas, models with ANOVA treatment for the dissolution data.

Zero-order equation (Wagner, 1969) is followed when the drug dissolution from FNM matrix layer is without disaggregate of the polymer and drug is released slowly in controlled manner. The following equation is adopted:

$$Q=Q_{0}+k_{0}t$$

Where *Q* represents the amount of drug dissolved in time *t, Q*_0_ is the initial amount of the drug in the solution and *k*_0_ is the zero order release constant expressed in units of concentration/time.

First-order equation (Gibaldi and Feldman, 1967; Wagner, 1969) is followed for the release of the drug from the matric and can be expressed by the first order release kinetics equation:

$$InQ=InQ_{0}+k_{1}t$$

where *k*_1_ is the first order rate constant and *t* is the time.

Higuchi equation (Higuchi, 1961) is followed when matrix is swelling and drug release is affected by change in the surface area. Higuchi equation defines a linear dependence of the active fraction released per unit of surface (*Q*) on the square root of time and can be expressed as

$$Q=k_{H}t^{1/2}$$

Where *Q* is the amount of drug release at time *t* and *k*_H_ is the Higuchi release constant.

Hixson–Crowell equation (Hixson and Crowell, 1931) was used to calculate the data:

$$Q^{1/}{{}^{3}}_{0}-Q^{1/3}{}_{t}=k_{s}t$$

Where *Q*_0_ is the initial amount of drug in the matrix tablet, *Q*_t_ is the amount of drug remaining in the dosage form at time *t*, and *k*_s_ is a constant incorporating the surface/volume ratio.

### Bioadhesion Studies

A modified balance method used for determining the *ex vivo* mucoadhesive strength (Shidhaye and Saindane, 2009; Shaikh et al., 2012). Fresh goat mucosa was obtained and used within 2 h of slaughter. The mucosal membrane separated by removing underlying fat and loose tissues. The membrane was washed with distilled water and then with 0.1N HCl at 37°C. The mucosa was cut into the pieces and washed. A piece of mucosa was tied to the Teflon piece, which was kept in beaker filled with HCl pH 1.2, at 37°C ± 1°C. The Teflon piece was tightly fitted into a glass beaker so that it just touched the mucosal surface. The bioadhesive tablet was stuck to the lower side of a pan. The two sides of the balance made equal before the study. A weight of 5 g was kept in the right-hand pan, which lowered the pan along with the tablet over the mucosa. The balance was kept in this position for 5 min to provide contact time for bioadhesion. The weight was removed. The water (equivalent to weight) was added slowly drop by drop with an infusion set to the left-hand pan until the tablet detached from the mucosal surface. The mucoadhesive strength of the bioadhesive tablet is calculated by formula

$$\mathrm{Detachment}\mathrm{stress}({dyne/cm}^{2})=m.g/A$$

Where, m = the weight added to the balance in gram, g = acceleration due to gravity taken as 980 cm/sec^2^, A = area of tissue exposed and is equal to πr^2^ (*r*-the radius of the circular hole in the aluminum cap).

### Bioadhesion Retention Time

The *ex vivo* bioadhesion time studies were performed (in triplicate) after application of tablets on freshly cut goat stomach mucosa. The mucosa was fixed on a glass slide using adhesive tape and kept in a slanting position in the beaker. A side of each tablet was wetted with 50 μl fluid and was attached to the mucosa by applying a light force with a fingertip for 30 s. The beaker was filled with 250 ml of simulated gastric fluid and kept at 37°C; a stirring rate of 100 rpm was applied. Tablet behavior and mucoadhesive time were monitored until complete detachment or dissolution occurred.

### Stability Study

Gastro retentive tablets of venlafaxine hydrochloride formulated in the present study were subjected to accelerated stability studies in aluminum/aluminum pouch pack. The tablets of check point batch F7 were charged for accelerated stability studies at 40°C and 75% RH for a period as prescribed by ICH guidelines for 3 months in a stability chamber. Bioadhesive retention time, mucoadhesive force, and drug dissolution profile of exposed sample was carried out after every month (Cartensen, 1995).

## Results and Discussion

Venlafaxine hydrochloride an antidepressant drug was formulated in to bilayer tablet comprising immediate release layer and sustained release layer to achieve loading dose and maintenance dose, respectively, and to give large duration of action. Total of nine batches were prepared for sustained release layer and six batches for immediate release layer. All the formulations were subjected to pharmacotechnical evaluation. The drug content of all formulations of sustained release layer varied between 98.13 and 102.45% w/w with mean ± SD as 100.29 ± 0.5%. Tablet weights of sustained release layer varied between 389.1 and 401.8 mg (395.45 ± 2.2 mg mean value), and thickness between 5.9 and 6.2 mm (6.05 ± 0.1 mm). All the tablets exhibited friability values ranging between 0.220 and 0.399%w/w (0.30 ± 0.14%), far less than the limit of 1% w/w. Marginal variation in tablet hardness and friability could be attributed only to the random causes, but not to the matrix composition. This absence of any significant inter- and intra-batch variability in tablet hardness, friability and thickness, ruled out any possibility of any change in compression pressure, and consequently in drug dissolution. All the immediate release layers disintegrated in <1 min. All six batches of immediate layer showed thickness, friability, weight variation, and content uniformity in acceptable limits. The batch B6 containing 10%w/w crosscarmellose was selected as an optimized formulations due to its minimum friability (0.01 ± 0.02%) and disintegration time 5 s.

Bioadhesion studies showed that with an increase in the amount of either polymer (Xanthan Gum, Carbopol, or HPMC). Bioadhesion strength increases due to hydration of polymer by hydrated mucous layer, resulting in reduced glass transition temperature and increased uncoiling along with an increased mobility of polymer chains. This tends to increase the adhesive surface for maximum contact with mucin and flexibility for interpenetration with mucin. Although the maximum value of bioadhesive strength was attained at the highest levels of all the polymers, yet the effect of selected polymers at selected levels with FNM was found to be almost similar without significant difference between the batches F1–F9 and F10–F12. The detachment force depicts the change in bioadhesive strength of tablets with a change in the polymer level(s).

Bioadhesive retention time for all tablets was found to be between 19 and 24 h. FNM has very good bioadhesive and combination of FNM with other polymer also showed a good bioadhesive retention time. As the percentage of fenugreek is decreased, the bioadhesive retention time is also decreased. Combinations of FNM with HPMC and Carbopol have high retention time.

**FIGURE 1:**
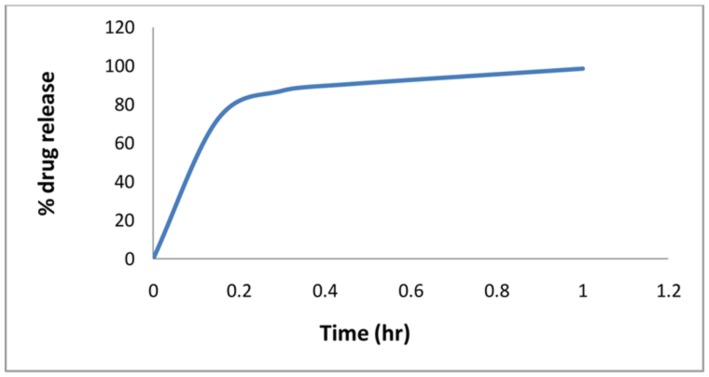
***In vitro* drug release profile for Immediate drug release layer (B6)**.

The *in vitro* dissolution studies were carried for immediate as well as sustained release layers. All six batches of immediate release layer formulations showed almost similar drug release patten. The **Figure 1** shows drug release pattern for batch B6.

**FIGURE 2:**
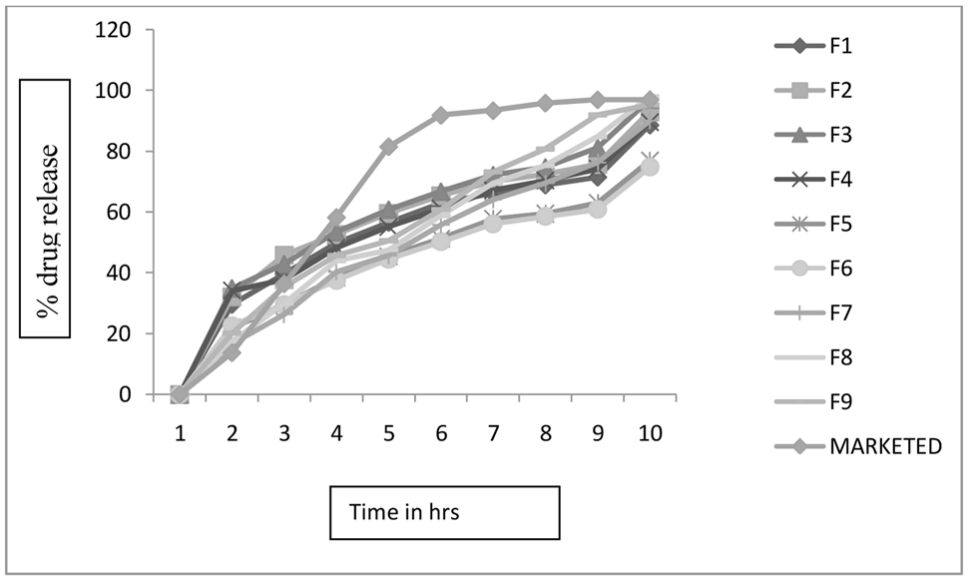
***In vitro* drug release profile for all batches of sustained release**.

All factorial as well as non-factorial batches of sustained release layer were subjected to *in vitro* dissolution studies. In the present studies, non-factorial batches containing only HPMC, Carbopol, and Xantham Gum individually were prepared to compare and study drug release pattern with factorial batches containing combination of these polymer with FNM. The **Figure 2** depicts the comparative drug release profile for all optimization batches along with marketed product.

All the polynomial equations were found to be statistically significant (*P* < 0.01), as determined using ANOVA, as per the provision of Design Expert software. The polynomial equations comprise the coefficients for intercept, first-order main effects, interaction terms, and the higher order effects. The sign and magnitude of the main effects signify the relative influence of each factor on the response.

**FIGURE 3:**
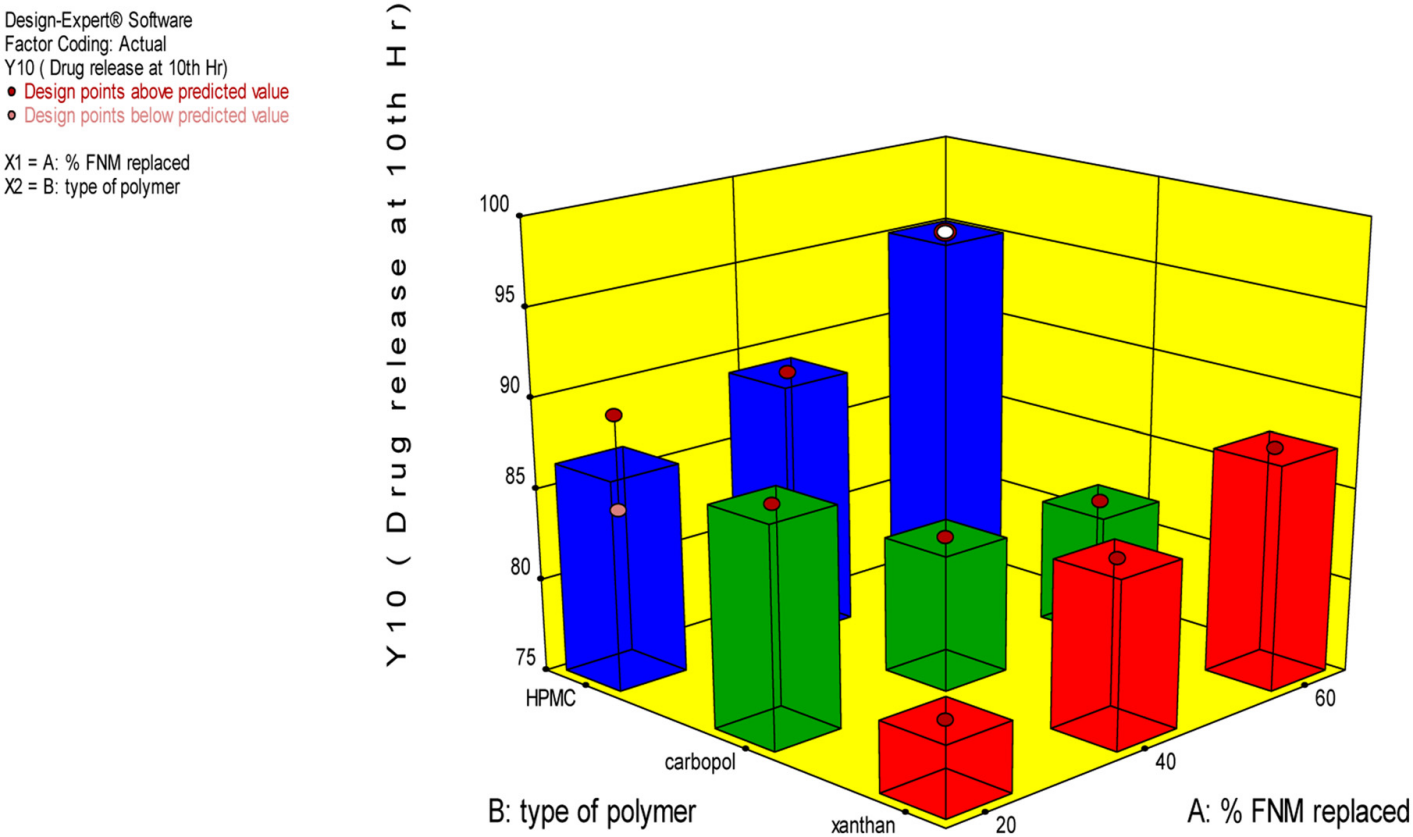
**3-D Graph for effect of independent variables on Y10**.

**FIGURE 4:**
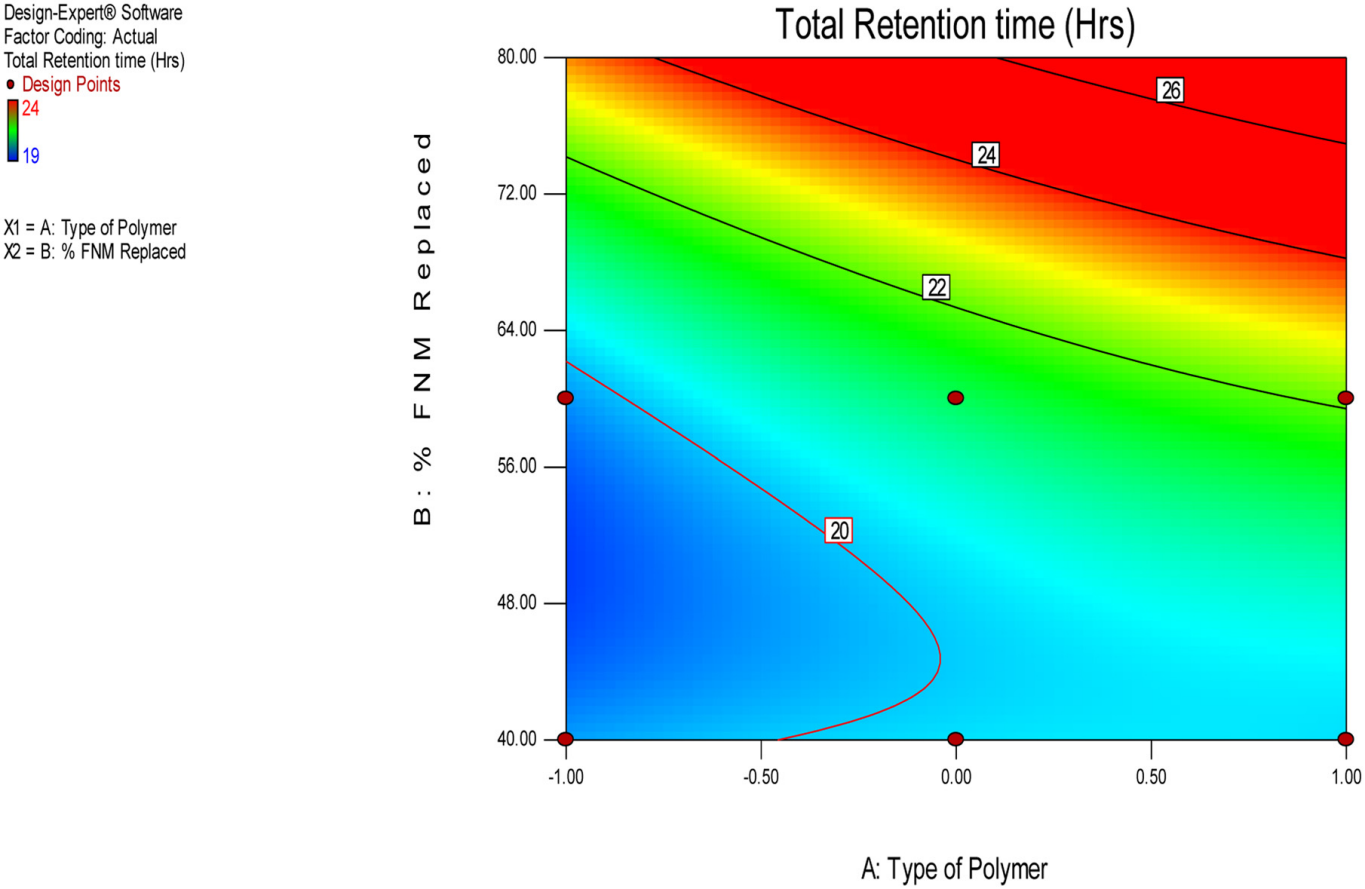
**Contour plot for effect of independent variables on the Total Retention Time**.

**FIGURE 5:**
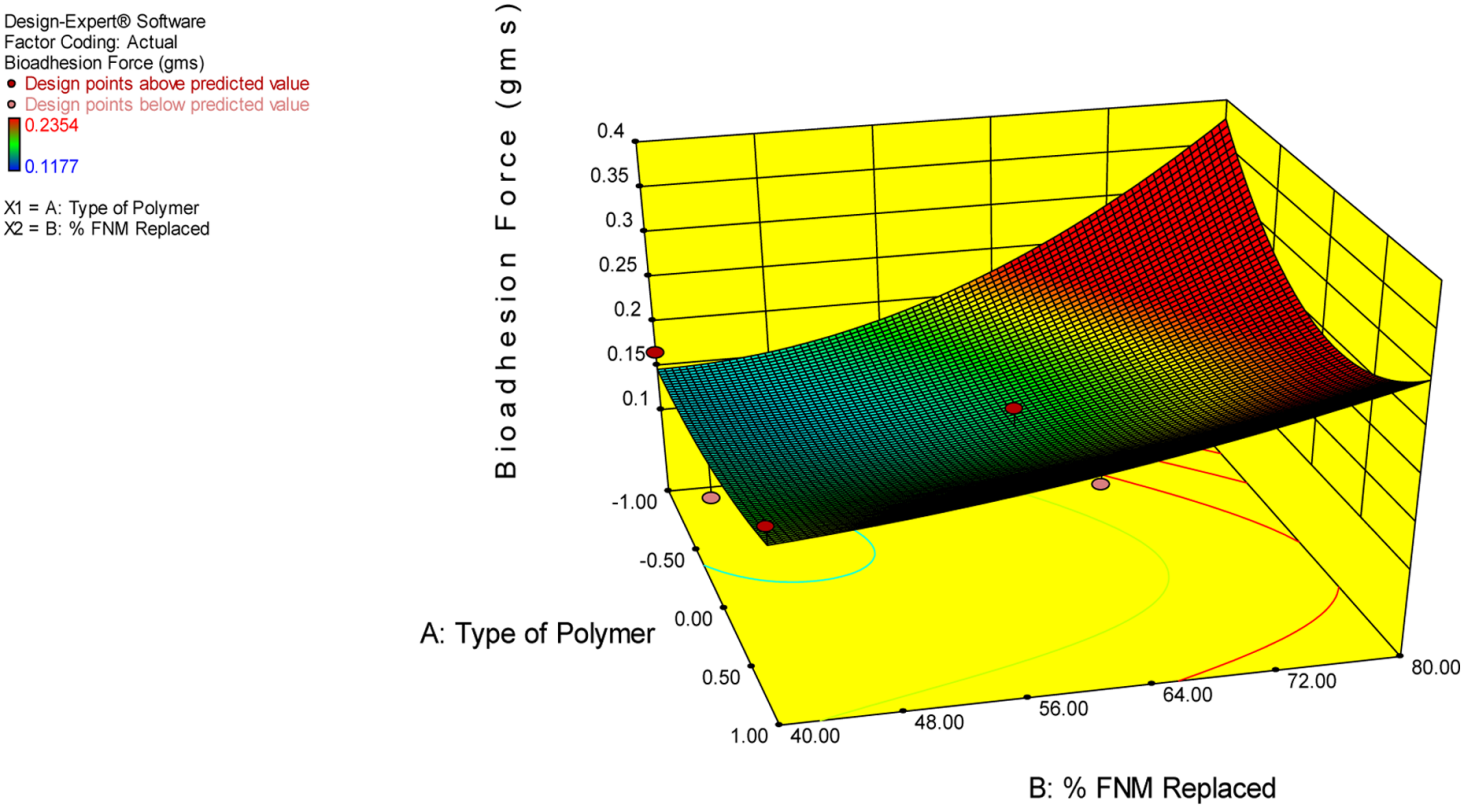
**Contour plot for effect of independent variables on the Bioadhesion Force**.

The independent variable, Y10, Bioadhesion force, and bioadhesion Retention Time were compared statistically using Design Expert software and polynomial equation was derived. The **Figure 3** depicts the effect of polymer and type of polymer used in combination with FNM at different level. From Eqs 2–4, it can be concluded that, Xanthan Gum has a predominant effect on drug release, as compared to Carbopol and HPMC. Xanthan Gum has a negative effect on the amount of drug release Whereas, Carbopol and FNM combination does not sustain drug release significantly. **Figure 3** depicts the response surface plot, showing the influence of HPMC, Xanthan Gum, and Carbopol-934P on Y10. The surface plot shows that Y10 varies as the concentration of the three polymers changes. From the contour plot (**Figures 4** and **5**), it can be concluded that all polymers at the selected levels with FNM significantly affect the bioadhesive strength and retention time. **Figure 5** presents the corresponding contour plot, showing the relationship between various levels of the three polymers.

From this graph and the derived polynomial equation it can be concluded that FNM: HPMC at 20:80 ratio could sustain drug release effectively till 10th hour as compared to carbopol and xanthan gum.

(2) $$Y10=86.01-1.71X1-2.39X2-3.00X1X2-0.{56X1}^{2}-2.08{X2}^{2}+1.{28X1}^{2}X2+5.{02X1X2}^{2}-{X1}^{2}{X2}^{2}(R^{2}\;=\;0.997,P\;<\;0.05)$$

Similarly, bioadhesion force and bioadhesion retention time have also shownsignificant effect based on combination of FNM with studied polymers. A goodbioadhesion retention time for tablet was observed when higher amount of HPMC orcarbopol was incorporated in the tablet (**Figure 4**). A satisfactory result and most optimized result in terms of bioadhesion force and retention wasobtained when FNM was replaced by 20% of its weight with HPMC (**Figure 5**).

(3) $$\mathrm{Bioadhesion}\mathrm{Force}=+0.299-0.014X1-7.19X2+6.13X1X2+0.{016X1}^{2}+8.{19X2}^{2}(R^{2}\;=\;0.877,P\;=\;0.065)$$

(4) $$\mathrm{Bioadhesion}\mathrm{Retention}\mathrm{Time}=+29.11-1.83X1-0.408X2+0.055X1X2-1.{66X1}^{2}+4.{58X2}^{2}(R^{2}=0.784,P=0.0925)$$

Dissolution parameters, shows that the value of *n* varies between 0.3963 and 0.6936, delineating non-Fickian release behavior (**Table 4**). The values of *n* show increasing trend with increase in HPMC content, However, the release of carbopol and Xanthan Gum is very slow and *n* seems to bear a nonlinear relationship. It shows a rising trend in the values of *n* as the content of FNM is increased with significant increase at the highest levels. The values of *k* followed a declining trend with increase in the amount of either polymer. Relatively much higher magnitude of *k1* clearly shows that the drug release was predominantly Fickian diffusion, with the contribution of polymer relaxation as nearly negligible. The overall rate of drug release tended to decrease with increase in concentration of Xanthan Gum and carbopol. Similarly, the values of Release at 10 h increased with increase in the polymer content of FNM : HPMC. The values of 12 h were found to enhance markedly from 3 to 8 h from low to high levels of both the polymers.

**Table 4:** Drug release kinetics data for all formulations.

Batch	Zero order		First order		Matrix		Peppas			Hix.Crow		Best fit model
	*R*^2^	*K*	*R*^2^	*K*	*R*^2^	*K*	*R*^2^	*K*	*N*	*R*^2^	*K*	
F1	0.614	9.17	0.905	-0.16	0.978	25.95	0.996	30.75	0.41	0.839	-0.043	Peppas
F2	0.585	9.751	0.935	-0.180	0.976	27.57	0.993	33.93	0.39	0.858	-0.047	Peppas
F3	0.637	10.100	0.964	-0.197	0.982	28.47	0.994	34.52	0.39	0.899	-0.051	Peppas
F4	0.724	9.528	0.981	-0.178	0.991	26.68	0.988	31.60	0.41	0.936	-0.047	matrix
F5	0.877	8.312	0.988	-0.140	0.997	22.89	0.997	21.36	0.54	0.975	-0.038	Peppas
F6	0.8752	8.163	0.984	-0.136	0.997	22.49	0.996	21.65	0.52	0.972	-0.037	Matrix
F7	0.902	9.035	0.993	-0.163	0.983	24.76	0.990	17.44	0.69	0.979	-0.043	First order
F8	0.896	9.729	0.985	-0.196	0.979	26.67	0.988	18.77	0.69	0.977	-0.050	Peppas
F9	0.890	10.401	0.970	-0.249	0.980	28.55	0.989	21.48	0.655	0.9797	-0.0586	Peppas
MKT	0.928	14.969	0.979	-0.439	0.955	35.87	0.962	17.35	0.946	0.9804	-0.0946	Hix.Crow
Bilayer	0.573	11.555	0.959	-0.237	0.947	31.02	0.983	44.89	0.274	0.8991	-0.0603	Peppas

**FIGURE 6:**
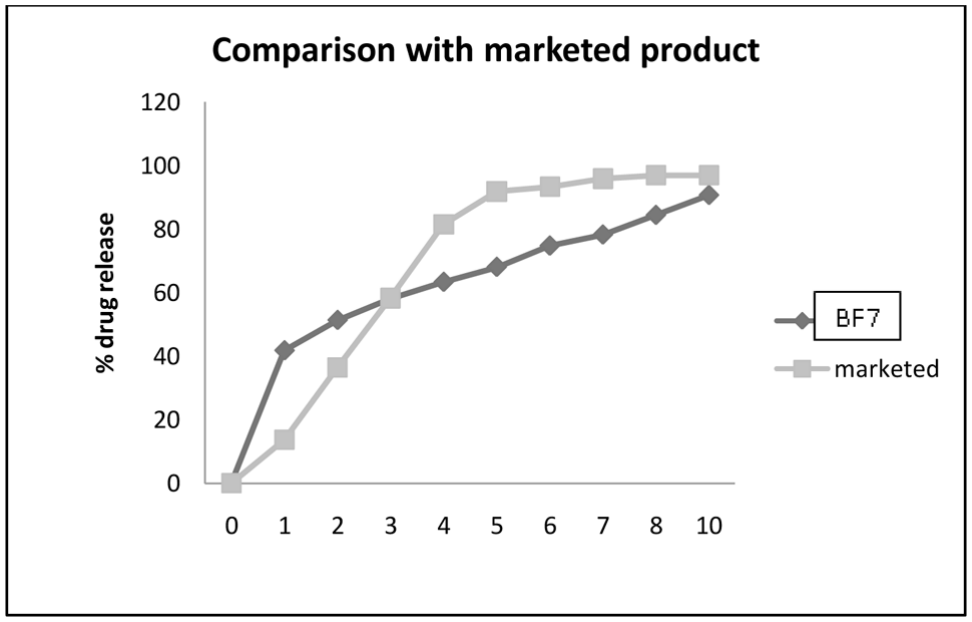
**comparison of marketed product and optimized batch BF7**.

The optimized immediate layer (B6) and sustained release layer (F6) were compressed to get bilayer tablet. The resultant bilayer tablet (BF7) was subjected to *in vitro* dissolution studies. The **Figure 6** indicates the initial burst effect due to immediate release layer of VFX and then slow release of the drug is maintained as per the Higuchi model of drug release kinetics till 12 h. It release up to 95 ± 0.5% drug after 12 h. Drug release profile of the optimized formulations was compared with the marketed brands of once-a-day formulations, Venlor XR each containing 75 mg of venlafaxine hydrochloride per tablet (**Figure 6**).

From the *in vitro* release studies of marketed formulation only 16% drug released in first hour. Drug release was found to follow zero order kinetics for marketed formulation. The formulation was found to provide sustained release for a period of 24 h with 96% drug being released in 12 h. The drug release profiles from the marketed formulations are shown in **Figure 6**. Our BF1 batch shows 40% of drug release in first hour and more than 80% of drug releases in 12 h. Our aim was to achieve 98–100% release in 24 h. Hence batch BF1 was chosen for stability. There was no change in the different physico-chemical parameters of the tablets at 40°C and 70% RH conditions of temperature and humidity.

## Conclusion

A bilayer tablets of VFX containing sustained release layer and immediate release layer were successfully formulated. Release was found to follow zero, krossmayer–peppas, and Hixon–crowel models. All the formulation batches tested for physical parameters like weight variation, hardness, friability and drug content, all were found to be within the USP limits. The optimized formulations were found to be stable at all the stability conditions. During stability studies, no significant variation (1–4%) in drug release was observed, indicating that formulation batch BF7 was stable over the chosen condition for 3 months. The optimized formulation BF1 showed better drug release profile as compare to marketed formulation Venlor XR. Combination of FNM and HPMC is an interesting polymer mixture for the preparation of SR matrix tablet because of good bioadhesive property, non toxicity and low cost of fenugreek and good binding capacity.

## Conflict of Interest Statement

The authors declare that the research was conducted in the absence of any commercial or financial relationships that could be construed as a potential conflict of interest.
